# The Amoebicidal Activity of Diferrocenyl Derivatives: A Significant Dependence on the Electronic Environment

**DOI:** 10.3390/molecules28166008

**Published:** 2023-08-11

**Authors:** Yanis Toledano-Magaña, Mario Néquiz, Lucía Margarita Valenzuela-Salas, Jessica J. Sánchez-García, Rodrigo Galindo-Murillo, Juan Carlos García-Ramos, Elena I. Klimova

**Affiliations:** 1 Escuela de Ciencias de la Salud, Universidad Autónoma de Baja California, Ensenada 22860, Mexico; 2 Unidad de Investigación en Medicina Experimental, Facultad de Medicina, Universidad Nacional Autónoma de México, Mexico City 06726, Mexico; manequiz@yahoo.com.mx; 3 Facultad de Ciencias de la Salud Unidad Valle de las Palmas, Universidad Autónoma de Baja California, Tijuana 22260, Mexico; 4 Facultad de Química, Universidad Nacional Autónoma de México, Ciudad Universitaria, Mexico City 04510, Mexico; heparina250ml@gmail.com (J.J.S.-G.); eiklimova@yahoo.com.mx (E.I.K.); 5 Department of Medicinal Chemistry, College of Pharmacy, University of Utah, Salt Lake City, UT 84112, USA; rodrigo.galindo@utah.edu

**Keywords:** amoebiasis, antiproliferative activity, metal-based drugs, diferrocenyl derivatives, electronic properties

## Abstract

Amoebiasis is the second leading cause of death worldwide associated with parasitic disease and is becoming a critical health problem in low-income countries, urging new treatment alternatives. One of the most promising strategies is enhancing the redox imbalance within these susceptible parasites related to their limited antioxidant defense system. Metal-based drugs represent a perfect option due to their extraordinary capacity to stabilize different oxidation states and adopt diverse geometries, allowing their interaction with several molecular targets. This work describes the amoebicidal activity of five 2-(*Z*-2,3-diferrocenylvinyl)-4X-4,5-dihydrooxazole derivatives (X = H (**3a**), Me (**3b**), iPr (**3c**), Ph (**3d**), and benzyl (**3e**)) on *Entamoeba histolytica* trophozoites and the physicochemical, experimental, and theoretical properties that can be used to describe the antiproliferative activity. The growth inhibition capacity of these organometallic compounds is strongly related to a fine balance between the compounds’ redox potential and hydrophilic character. The antiproliferative activity of diferrocenyl derivatives studied herein could be described either with the redox potential, the energy of electronic transitions, logP, or the calculated HOMO–LUMO values. Compound **3d** presents the highest antiproliferative activity of the series with an IC_50_ of 23 µM. However, the results of this work provide a pipeline to improve the amoebicidal activity of these compounds through the directed modification of their electronic environment.

## 1. Introduction

Amebiasis is a silent killer that remains a public health problem in low-income countries [1] but has recently re-emerged with great force in developed countries, mainly associated with travelers visiting endemic areas and immigration [2]. The disease is caused by the protozoan *Entamoeba histolytica*, which settles in the intestine without causing symptoms in most cases. However, in 10% of cases, the parasite can invade other tissues, mainly the liver, generating an amebic liver abscess (ALA) [3]. Despite being responsible for more than 55,000 deaths worldwide from amoebic colitis [4] and the second leading cause of death worldwide from parasitic diseases [5], it still does not appear on the list of neglected tropical diseases (NTDs) published by the World Health Organization [6].

Many studies have focused on generating a vaccine; however, the effort has been unsuccessful so far [4]. However, progress has been made in identifying different therapeutic targets that can eliminate the pathogenicity of the parasite or eliminate it completely [7]. One of the most favorable strategies is to take advantage of the high sensitivity of the trophozoite to the redox imbalance produced by the excessive production of reactive oxygen species [8]. This strategy seems promising because *E. histolytica* has a minimal antioxidant defense system that depends on cysteine and thioredoxin [9].

To exploit the deficient antioxidant defense of trophozoites, one of the best options is to use metal-based compounds. These compounds can stabilize different oxidation states, adopt different geometries, and modulate the reactivity and exchange of the ligands that make up their coordination sphere, enabling their combination with multiple biological targets that have been highly effective in eliminating different parasites [10,11,12,13,14].

On the other hand, 4,5-oxazole derivatives are essential due to their potential application in material sciences [15], biotechnology [16,17], agriculture [18], and medicine, including antibacterial [19], antioxidant [20], anticancer [21], antidiabetic [22], antiparasitic [23], and anti-inflammatory activities [24], among others. The synthesis of 4,5-dihydrooxazoles is performed using the cyclocondensation of 2-aminoalcohols with the derivatives of carboxylic acids or carbaldehydes [25,26]. The oxazole structure can be found in commercial compounds currently used in therapy, like linezolid, furazolidone, toloxatone, oxaprozin, and ditazole.

It is known that ferrocenyl compounds exhibit important biological properties [27]. Specifically, for amoebiasis, different redox-active compounds with remarkable amoebicidal activity in vitro and in vivo have been evaluated [28,29,30,31,32]. Among those, ferrocene derivatives are one of the most studied due to their high stability and enormous capacity to participate in redox reactions, stabilizing both Fe(II) and Fe(III) oxidation states [33,34,35,36,37]. The synergy of oxazole molecules with ferrocene units could have interesting biological responses due to their favorable practical properties, e.g., thermal stability, electrical conductivity, and nonlinear optical effect. In this work, 2-(*Z*-2,3-diferrocenylvinyl) 4-X-4,5-dihydrooxazole derivatives were obtained using the condensation of 2,3-diferrocenylcyclopropenylium salts with the derivatives of 1,2-aminoalcohols. Furthermore, their antiproliferative activity against HM1: IMSS *Entamoeba histolytica* trophozoite cultures and their dependence on the redox potential and the electronic environment of the compounds are reported.

## 2. Results and Discussion

### 2.1. Characterization

**Figure 1:**
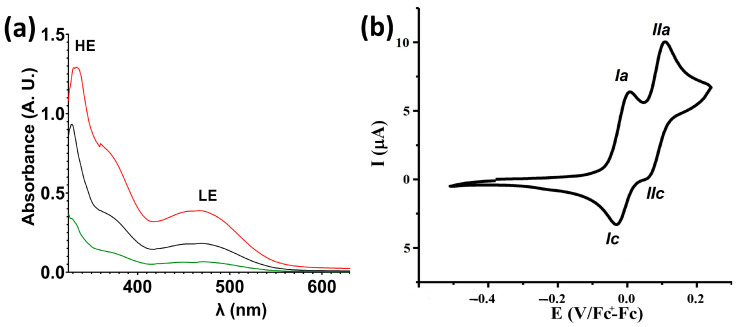
(**a**) Electronic spectra and (**b**) voltammogram of compound **3c** (2-(*Z*-1,2-diferrocenylvinyl)-4-isopropyl-4,5-dihydrooxazole.

The electronic spectra of **3c** (2-(*Z*-1,2-diferrocenylvinyl)-4-isopropyl-4,5-dihydrooxazole) is shown in Figure 1a. The transition observed at 329 nm (high energy, HE) corresponds to the transition from HOMOs located on the CP and π-conjugated bridge to the LUMOs corresponding to the π-conjugated bridge, with some contribution from the acceptor and d-orbitals of the metal. On the other hand, the maximum observed at 464 nm corresponds to the low-energy transition (LE) involving the nonbonding HOMO orbitals predominantly located on the metal to the lowest unoccupied orbital, mainly formed by the anti-bonding p orbitals of the linker and/or acceptor moiety. The assignments were based on those described for ferrocene derivatives and corresponded with the results obtained by DFT described in the following paragraphs [38]. Similar results were obtained for the rest of the compounds and compiled in Table 1.

**Figure 2:**
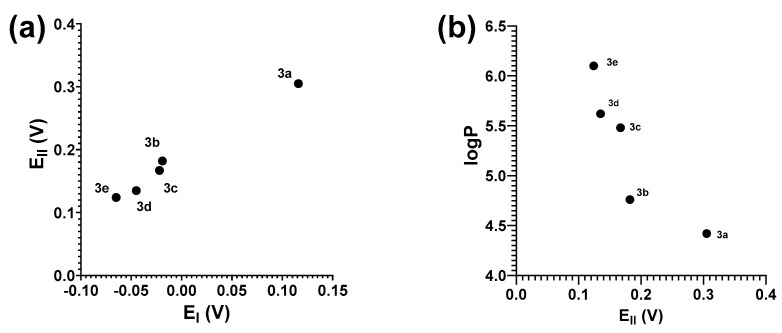
Relationship between (**a**) the two redox transformations and (**b**) the redox and hydrophobic response observed for diferrocenyl derivatives.

On the other hand, Figure 1b shows the electrochemical behavior of compound **3c** in acetonitrile. Two oxidation (Ia, IIa) and two reduction (Ic, IIc) processes with half-wave potential values E(I) = −0.022 V and E(II) = 0.164 V were observed. The electronic communication between both Ferrocene units was evaluated using the ΔE(II–I) to calculate the comproportionation constant (K_com_) [39]. According to the K_com_ value 1563, the mixed-valence compound is a Class II compound in the Robin–Day classification with slight electronic delocalization between the two redox centers. This slight communication between iron ions allows the identification of two independent and sequential redox processes. It confirms the absence of chemical or adsorption processes associated with redox transformation (Figure 1b). Similar results were found for compound **3e** (Table 1). The relationship between redox potential values found for compounds **3a**–**e** follows the equation E_II_(V) = 1.01E_I_(V) + 0.1897 (R^2^ = 0.9894, F = 280.7, Figure 2a). The electrochemical parameters for compounds **3c** and **3e** are comparable with those observed for compounds **3a**, **3b**, and **3d** previously reported [37]. 

The efficiency of electronic communication strongly depends on the substituent inductive effect. As the most potent inductive effect, it is more challenging to produce the oxidation of the metal centers, as shown by the comparison of redox potential with similar compounds previously reported [40,41]. Compound **3a** shows E(I) = 0.116 V and E(II) = 0.305 V, while changing the 4,5-dihydrooxazole moiety for an imidazoline [40] or a 4,5-dihydro-1,3-thiazole [41] shifts the redox potential to higher positive values. The redox potential values for 2-(*Z*-1,2-diferrocenylvinyl)-imidazoline are E(I) = 0.250 V and E(II) = 0.437 V [40] and for 2-(*Z*-1,2-diferrocenylvinyl)-4,5-dihydro-1,3-thiazole they are E(I) = 0.251 V and E(II) = 0.408 V [41]. 

The logP values of the organometallic compounds were obtained by the reversed-phase thin-layer chromatography (RP-TLC) method. Huber and co-workers [42,43] reported the relationship between partition coefficient and retention factor derived from R*_f_* according to equation

logP = aR_M_ + b

The logP values obtained for the compounds studied herein are compiled in Table 1. The values are within the range of 4.4 to 6.1, confirming its low water solubility. The obtained values agree with those reported for other ferrocene derivatives theoretically or experimentally determined [44,45,46,47]. 

There is no linear inverse relationship between the redox behavior and the lipophilic character of the diferrocenyl derivatives. However, it is clear that as hydrophobicity decreases, the redox potential values for organometallic compounds increase (Figure 2b). 

### 2.2. Computational Chemistry

**Figure 3:**
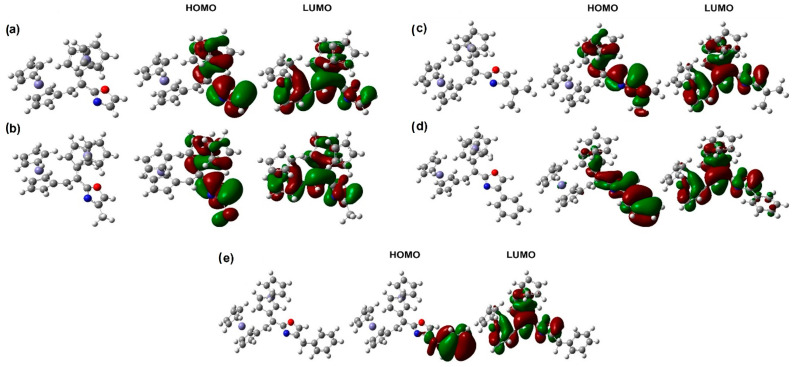
HOMO and LUMO molecular orbitals with an isosurface value of 0.2 for compounds **3a**–**e**. The molecular structure of the compound, the HOMO orbital, and the LUMO orbital are shown from left to right. (**a**) compound **3a**, (**b**) compound **3b**, (**c**), compound **3c**, (**d**) Compound **3d**, and (**e**) compound **3e**.

A DFT study performed further analysis of the electronic distribution of compounds **3a**–**e**. The optimized structure of compound **3a** was obtained from the crystallographic data reported by Sánchez-Garcia et al. [36] using an M06-2× level of theory and the base 6-311G++ (2d, 2p). The optimization parameters were employed to treat the rest of the molecules following the same procedure. Figure 3 shows that the higher the inductive effect of the oxazole substituent, the higher the ferrocene unit’s participation in the HOMO orbital. A clear example can be found comparing the HOMO orbital distribution in compounds **3b** and **3e**. In compound **3b**, the HOMO orbital is distributed in oxazole and one of the ferrocenyl units. In contrast, the HOMO in compound **3e** is exclusively located in the benzyl moiety without the participation of ferrocenes (Figure 3e). On the other hand, the LUMO orbital is mainly situated in the diferrocenylvinyl and the 4,5-dihydrooxazole units, with minimal (Figure 3d) or without (Figure 3b,c,e) participation of the substituent of 4,5-dihydrooxazole. The HOMO–LUMO distribution described above agrees with the electronic transition assignment for the organometallic compounds (Table 1). 

**Figure 4:**
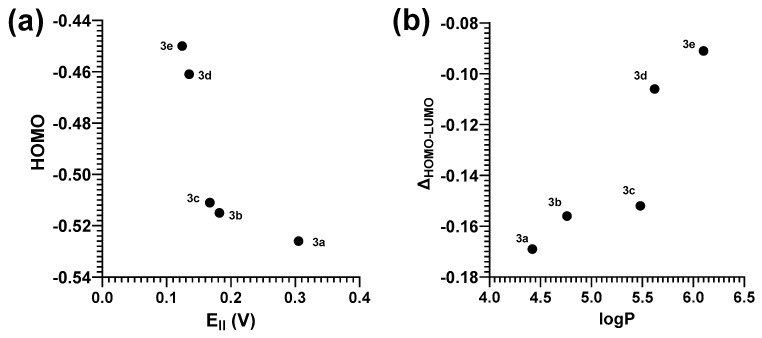
Relationship between theoretical parameters and experimental properties of 2-(*Z*-1,2-diferrocenylvinyl)-4,5-dihydrooxazole derivatives: (**a**) redox potential vs. HOMO and (**b**) logP vs. Δ_HOMO–LUMO_.

Electronic parameters obtained from DFT have been used to describe the experimental properties of diferrocenyl derivatives. No linear relationship was found; nevertheless, the HOMO and Δ_HOMO–LUMO_ could explain the redox potential and hydrophobic properties except for compound **3a** for the former and compound **3c** for the latter (Figure 4). Without the outliner compounds, the linear relationships follow the expressions:

E_II_ = −0.7935 HOMO − 0.2323 R^2^ = 0.9668, F = 58.30

logP = 20.03 Δ_HOMO–LUMO_ + 15.00 R^2^ = 0.976, F = 83.25

### 2.3. Amoebicidal Activity

The amoebicidal capacity of compounds **3a**–**e** was determined on trophozoites of *Entamoeba histolytica* HM1:IMSS strain cultures and is compiled in Table 2. The compound with a non-substituted oxazole ring (**3a**), considered a “parent compound,” presents poor antiproliferative activity with IC_50_ = 1000 μM. Incorporating an alkyl substituent to **3a** (compound **3b**, IC_50_ = 100 μM) produces a 10-fold increase in the amoebicidal activity; however, a negative steric contribution appears when −iPr is the substituent (compound **3c**, IC_50_ = 1891.9 μM). A phenyl ring as a substituent for an oxazole ring (the compound **3d**) increases the amoebicidal potency more than 40 times (23 μM) compared with the parent compound **3a**, but incorporating a benzyl unit (**3e**) produces an amoebicidal activity increase of 2.5 times. Therefore, a balance between inductive and steric effects should exist to efficiently contribute to electronic communication and amoebicidal activity. 

A close relationship was found between the amoebicidal activity and the electronic properties of the organometallic compounds **3a**, **3b**, and **3d**. The oxazole ring substituents’ electron donor capacity modulates the redox potential, similar to the metal ions’ electronic transition. The electronic communication between the Fe(II) ions of the ferrocene units will modulate the inductive effect of the oxazole substituents, which in turn, depends hugely on the linker structure [38]. This also affects the interaction of the molecules with the solvent and defines its lipophilic character. In this series, the redox potential becomes more positive as the electron donor capacity of the substituent becomes more outstanding. A similar response was observed in the electronic transition, as the higher the electron withdrawal, the lower the energy required to undergo the electronic transition of the LE process. 

**Figure 5:**
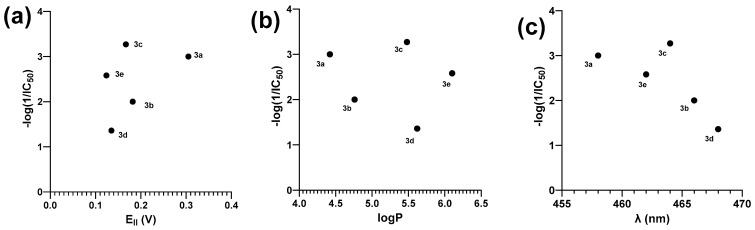
Relationship between the antiproliferative activity of organometallic derivatives and their physicochemical properties. (**a**) Redox potential, (**b**) logP, and (**c**) electronic transitions of 2-(*Z*-1,2-diferrocenylvinyl)-4,5-dihydrooxazole derivatives vs. the −log (1/IC_50_).

The highest antiproliferative activity was observed with compounds with the lowest redox potential vs. Fc/Fc+ of the series, either using E(I) or E(II) values (Figure 5a) or with the highest lambda value for LE electronic transition (Figure 5c). This result suggests that electronic communication between two redox centers in the electrochemical study is a significant factor in the amoebicidal activity of the diferrocenyloxazole derivatives studied in this work.

The above-described behavior agrees with the antiproliferative activity on amoeba trophozoites previously reported for the mono-ferrocenyl derivatives (5,6,6a,7-tetrahydrobenzo[*b*]naphtho [1,2-*e*][1,4]thiazepin-7-yl)ferrocene and (2,3,4-trimethoxy-5,6,6a,7-tetrahydrobenzo[*b*]naphtho [1,2-*e*][1,4]thiazepin-7-yl)ferrocene that show an IC_50_ = 7.5 and 12.6 µM with redox potential values of +0.005 V and +0.042 V, respectively [36]. However, significant modifications in the units linked to ferrocene considerably change the electronic environment and the inductive effect of the substituents of those linkers. Different examples could be found in the complete revision performed by Kaur and co-workers [38]. This could explain why some mono-ferrocenyl compounds have shown increased amoebicidal potency when the main structure includes electron donor groups as substituents. The incorporation of the methoxy group increases 2.5 to 35 times the amoebicidal activity of the family of compounds 4-(substituted aryl)-2-pyrrolidin-1-yl-6-ferrocenyl pyrimidine [34], 4-(substituted aryl)-6-ferrocenyl-2-piperidin-1-yl-pyrimidine [34], 4-(substituted aryl)-6-ferrocenyl-2-morpholin-1-yl-pyrimidine [35], and 5-ferrocenyl-3-(substituted aryl)-4,5-dihydropyrazole-1-carbothioamide [33]. Unfortunately, there is no information about the electrochemical behavior of these compounds. 

Although the redox processes for metronidazole and ferrocenyloxazole derivatives are entirely different, the final result will be the redox unbalance that leads to DNA damage and cell death in both cases. Metronidazole requires a nitro group reduction by the plasma-membrane enzyme pyruvate-ferredoxin oxidoreductase (EhPFOR), mainly located in the plasma membrane of the trophozoite [48], to generate the active species nitro radical anion [49]. On the other hand, the diferrocenyloxazole derivatives could participate in Fenton reactions through the oxidation of Fe^+2^ to Fe^+3^ by H_2_O_2_ [50,51,52,53], reactions that could proceed 103–104 times faster in the hydrophobic/hydrophilic interface than in bulk [54], enhancing the amoebicidal efficacy of ferrocenyl derivatives. Furthermore, ferrocenyl derivatives could inhibit the activity of thioredoxin, significantly affecting the antioxidant response of *E. histolytica* due to the fact that the protozoa lacks typical oxidative defense systems such as catalase, peroxidase, glutathione, and glutathione recycling enzymes, and the antioxidant response relies on cysteine and thioredoxin [9]. Figure 5b shows that compound **3d**, one of the most hydrophobic of the series, has the highest antiproliferative activity. 

Taken together, the results obtained in this work provide a pipeline for the rational design of metal-based amoebicidal agents. The hydrophobic and redox behaviors of compound **3d** exhibit the balance needed between the redox potential and hydrophobic character to increase the antiproliferative activity of this series. The ongoing work includes electron donor substituents on the benzene ring, taking compound **3d** as the new “parent compound”.

## 3. Materials and Methods

### 3.1. Synthesis and Characterization

All the solvents were dried according to standard procedures and were freshly distilled before use. Column chromatography was carried out on alumina (Brockmann activity III). The ^1^H and ^13^C NMR spectra were recorded on a Unity Inova Varian spectrometer (Varian, Inc, Palo Alto, CA, USA, 300 and 75 MHz) for solutions in CDCl_3_ with Me_4_Si as the internal standard. The IR spectra were measured on a FT-IR spectrophotometer (Spectrum RXI Perkin Elmer instruments, Waltham, MA, USA) using KBr pellets (Aldrich, St. Louis, MO, USA). The mass spectra were obtained on a Varian MAT CH-6 instrument (Varian, Inc, Palo Alto, CA, USA, EI MS, 70 eV). The elemental analyzer system LECO CHNS-900 (Leco, Corporation, St. Joseph, MI, USA) was used for elemental analyses.

The following reagents were purchased from Aldrich (St. Louis, MO, USA): triethyloxonium tetrafluoroborate, 1.0 M solution in dichloromethane; morpholine, 99%; piperidine, 99%; triethylamine, 99.5%; ethanolamine, 99.5+%; 2-amino-1-propanol, 98%; (*R*)-(−)-2-amino-2-phenylethanol, 98%; (*S*)-(−)-2-amino-3-phenylpropanol, 98%; 2-amino-3-methyl-1-propanol, 97; Al_2_O_3_ and RP-TLC silica gel 60 plates, F_254_.

Ethoxy(diferrocenyl)cyclopropenylium tetrafluoroborate was obtained from the 2,3-diferrocenylcyclopropenone in the presence of triethyloxonium tetrafluoroborate (1.0 M solution in dichloromethane) [55]. Diferocenyl(morpholino)cyclopropenylium tetrafluoroborate was obtained from ethoxy(diferrocenyl)cyclopropenylium tetrafluoroborate and morpholine in dichloromethane [56]. 

### 3.2. Reaction of 2,3-Diferrocenyl-1-morpholinocyclopropenylium Tetrafluoroborates (***1a***) with 1,2-Amino Alcohols (***2a***–***e***) (General Procedure)

**Scheme 1:**
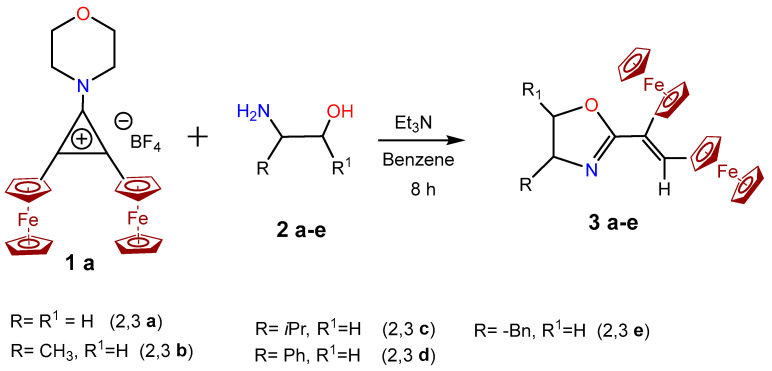
Reactions of the diferrocenylcyclopropenylium salts **1a** with 1,2-aminoalcohols **2a**–**e**.

1,2-Amino alcohols (**2a**, **2b**, **2c**, **2d**, **2e**, 5 mmol) and Et_3_N (1.0 mL) were added with stirring to the solution of 2,3-diferrocenyl-1-morpholinocyclopropenilium tetrafluoroborates **1a** (3 mmol) in dry benzene (70 mL, JT Baker, Phillipsburg, NJ, USA). After stirring for 8 h at 80 °C, the volatiles were removed in vacuo; chromatography of the residue on Al_2_O_3_ (hexane-ether, 4:1, JT Baker, Phillipsburg, NJ, USA) yielded compounds **3a**–**e** (Scheme 1).

**Figure 6:**
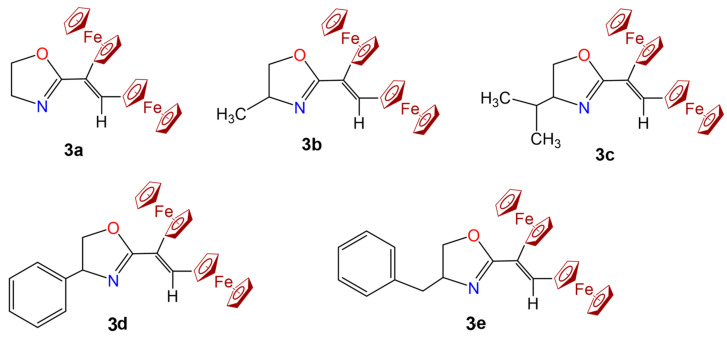
Structures of diferrocenylvinyloxazolines studied in this work. (**3a**) 2-(*Z*-2,3-diferrocenylvinyl)-4,5-dihydrooxazole; (**3b**) 2-(*Z*-1,2-diferrocenylvinyl)-4-methyl-4,5-dihydrooxazole; (**3c**) 2-(*Z*-1,2-diferrocenylvinyl)-4-isopropyl-4,5-dihydrooxazole; (**3d**) 2-(*Z*-1,2-diferrocenylvinyl)-4-phenyl-4,5-dihydrooxazole; (**3e**) 2-(*Z*-1,2-diferrocenylvinyl)-4-benzyl-4,5-dihydrooxazole.

Individual diferrocenylvinyloxazolines **3a**–**e** (Figure 6) were isolated using Al_2_O_3_ column chromatography (activity grade III, elution with hexane-ether 4:1). In the solid state, fine crystalline substances of orange color represent them. The E and Z enantiomers could be separated by a chromatographic column. In this work, only *Z*-enantiomers were employed, as described in the compound characterization. The structures of compounds **3a**–**e** were determined based on mass spectrometry, FT-IR, ^1^H, and ^13^C NMR spectroscopy, and elemental analysis. UV–vis spectra of **3a**–**e** were acquired using acetonitrile solutions at different concentrations in a Genesis 30 Spectrophotometer (Thermo Scientific, Waltham, MA, USA) from 325 to 1100 nm. Electrochemical measurements for compounds **3c** and **3e** were performed using conditions reported previously [37]. Briefly, acetonitrile solutions at different concentrations of the compound with tetrabutylammonium tetrafluoroborate (TBATFB) 0.1M as a supporting electrolyte, platinum disk as a working electrode, and Ag/AgNO_3_ as a reference in a potentiostat/galvanostat (EG&G PAR 273). The scan rate was 0.1 V/s, from open circuit potential to positive direction. Bubbling N_2_ deoxygenated the working solution before each measurement.

2-(*Z*-2,3-Diferrocenylvinyl-4,5-dihydrooxazole (**3a**) orange crystals, m.p. 123–124 °C. IR (KBr): ν 481, 535, 587, 659, 730, 813, 891, 827, 858, 947, 999, 1039, 1027, 1105, 1179, 1242, 1306, 1357, 1348, 1477, 1630, 1762, 2870, 2893, 3087, 3150 cm^−1^. ^1^H NMR [300 MHz, CDCl_3_]: δ 4.05 (5H, s, C_5_H_5_), 4.06 (2H, t, CH_2_, *J* = 9.0 Hz), 4.07 (5H, s, C_5_H_5_), 4.19 (4H, m, C_5_H_4_), 4.23 (2H, m, C_5_H_4_), 4.44 (2H, t, CH_2_, *J* = 9.0 Hz), 4.48 (2H, m, C_5_H_4_), 7.15 (1H, s, CH=). ^13^C NMR [75 MHz, CDCl_3_]: δ 50.42, 63.78 (2CH_2_), 69.14, 69.34 (2C_5_H_5_), 67.84, 69.19, 69.51, 70.50 (2C_5_H_4_), 79.60, 79.41 (2C_ipso_Fc), 136.94 (CH=), 126.30, 167.62 (2C). Anal. calcd. for C_25_H_23_Fe_2_NO: C, 64.55; H, 4.99; N, 3.01. Found: C, 64.17; H, 4.93; N, 3.01%. MS (El, 70 eV): *m*/*z* 465 [M]^+^. UV–vis (λ, nm): 330, 368, 458. E(I) = 116 mV, E(II) = 305 mV.
2-(*Z*-1,2-Diferrocenylvinyl)-4-methyl-4,5-dihydrooxazole (**3b**) orange powder, m.p. 127–129 °C. IR (KBr): ν 471, 645, 732, 749, 810, 849, 971, 1006, 1041, 1163, 1241, 1277, 1315, 1340, 1410, 11,457, 1475, 1588, 1617, 1724, 2248, 2852, 2922, 3086, 3462 cm^−1^. ^1^H NMR [300 MHz, CDCl_3_]: δ 1.42 (3H, d, CH_3_, *J* = 6.6 Hz), 3.93 (1H, t, CH_2_, *J* = 7.8 Hz), 4.08 (10H, s, 2C_5_H_5_), 4.19 (4H, m, C_5_H_4_), 4.20 (1H, m, C_5_H_4_), 4.23 (4H, m, C_5_H_4_), 4.36 (1H, m, CH), 4.47 (2H, m, C_5_H_4_), 4.54 (1H, dd, CH_2_, *J* = 7.8, 9.3 Hz),7.14 (1H, s, CH=). ^13^C NMR [75 MHz, CDCl_3_]: δ 21.73 (CH_3_), 61.82 (CH), 67.75 (CH_2_), 69.27, 69.35 (2C_5_H_5_), 69.06 (2C), 69.58, 70.21, 70.44, 70.62, 70.79, 73.68 (2C_5_H_4_), 80.55, 80.59 (2C_ipso_Fc), 134.20 (CH=), 123.78, 165.35 (2C). Anal. calcd. for C_26_H_25_Fe_2_NO: C, 65.17; H, 5.26; N, 2.92. Found: C, 65.36; H, 5.35; N, 3.16%. MS (El, 70 eV): *m*/*z* 479 [M]^+^. UV–vis (λ, nm): 331, 372, 466. E(I) = −19 mV, E(II) = 182 mV.
2-(*Z*-1,2-Diferrocenylvinyl)-4-isopropyl-4,5-dihydrooxazoline (**3c**) orange powder, m.p. 85–87 °C. IR (KBr): ν 478, 707, 738, 800, 811, 886, 929, 972 998, 1038, 1056, 1105, 1174, 1189, 1244, 1295, 1348, 1381, 1449, 1468, 1607, 1630, 1703, 2823, 2905, 3085, 3092 cm^−1^. ^1^H NMR [300 MHz, C_6_D_6_]: δ 0.83 (3H, d, CH_3_, *J* = 6.6 Hz), 1.11 (3H, d, CH_3_, *J* = 6.6 Hz), 1.67 (1H, m, CH), 3.74 (1H, m, CH), 3.82 (1H, dd, CH_2_, *J* = 6.9, 8.7 Hz), 4.02 (1H, dd, CH_2_, *J* = 7.2, 8.7 Hz), 3.87 (5H, s, C_5_H_5_), 3.98 (2H, m, C_5_H_4_), 4.10 (1H, m, C_5_H_4_), 4.13 (5H, s, C_5_H_5_), 4.15 (1H, m, C_5_H_4_), 4.27 (2H, m, C_5_H_4_), 4.80 (1H, m, C_5_H_4_), 4.88 (1H, m, C_5_H_4_), 7.62 (1H, s, CH=). ^13^C NMR (75MHz, C_6_D_6_): δ 19.05, 19.73 (2CH_3_), 33.96, 68.0 (2CH), 68.05 (CH_2_), 69.73, 70.01 (2C_5_H_5_), 69.44, 69.46, 69.89, 70.56, 70.98, 71.33, 71.67, 73.97 (2C_5_H_4_), 81.55, 81.60 (2C_ipso_Fc), 134.43 (CH=), 124.61, 164.51 (2C). Anal. calcd. for C_28_H_29_Fe_2_NO: C, 66.30; H, 5.76; N, 2.76. Found: C, 66.46; H, 5.54; N, 2.80. MS (El, 70 eV): *m*/*z* 507 [M]^+^. UV–vis (λ, nm): 329, 368, 464. E(I) = −22 mV, E(II) = 167 mV.
2-(*Z*-1,2-Diferrocenylvinyl)-4-phenyl-4,5-dihydrooxazoline (**3d**) orange powder, m.p. 153–154 °C. IR (KBr): ν 480, 494, 624, 698, 815, 898, 930, 999, 1031, 1049, 1105, 1183, 1184, 1242, 1256, 1281, 1331, 1377, 1449, 1477, 1491, 1603, 1711, 1741, 2851, 2923, 3092 cm^−1^. ^1^H NMR [300 MHz, CDCl_3_]: δ 4.04 (5H, s, C_5_H_5_), 4.07 (5H, s, C_5_H_5_), 4.18 (1H, t, CH, *J* = 8.7 Hz), 4.20 (3H, m, C_5_H_4_), 4.24 (3H, m, C_5_H_4_), 4.27 (1H, m, C_5_H_4_), 4.50 (1H, m, C_5_H_4_), 4.74 (1H, dd, CH_2_, *J* = 8.7, 9.9 Hz), 5.38 (1H, dd, CH_2_, *J* = 8.7, 9.9 Hz), 7.29 (1H, s, CH=), 7.38–7.40 (5H, m, C_6_H_5_). ^13^C NMR [75 MHz, (CD_3_)_2_CO)]: δ 67.83 (CH), 67.90 (CH_2_), 69.41, 69.48 (2C_5_H_5_), 69.28, 69.86, 70.16, 70.44, 70.55, 70.78, 70.88, 74.07 (2C_5_H_4_), 80.48, 80.64 (2C_ipso_Fc), 126.87, 127.62, 128.84 (C_6_H_5_), 135.04 (CH=), 123.39, 142.66, 166.45 (3C). Anal. calcd. for C_31_H_27_Fe_2_NO: C, 68.79; H, 5.03; N, 2.59. Found: C, 68.53; H, 5.02; N, 2.35%. MS (El, 70 eV): *m*/*z* 541 [M]^+^. UV–vis (λ, nm): 334, 370, 468. E(I) = −45 mV, E(II) = 135 mV.
4-Benzyl-2-(*Z*-1,2-diferrocenylvinyl)-4,5-dihydrooxazoline (**3e**) orange powder, m.p. 119–120 °C. IR (KBr): ν 482, 644, 695, 709, 734, 803, 815, 832, 878, 911, 955, 997, 1027, 1035, 1047, 1104, 1185, 1213, 1267, 1306, 1356, 1410, 1454, 1480, 1497, 1602, 1635, 1711, 1775, 1948, 2087, 2200, 2853, 2923, 3029, 3086, 3106 cm^−1^. ^1^H NMR [300 MHz, C_6_D_6_]: δ 2.63 (1H, dd, CH_2_, *J* = 8.1, 13.8 Hz), 3.13 (1H, dd, CH_2_, *J* = 6.0, 13.8 Hz), 3.80 (1H, t, CH_2_, *J* = 8.7 Hz), 3.88 (5H, s, C_5_H_5_), 3.93 (1H, t, CH_2_, *J* = 8.7 Hz), 3.98 (2H, m, C_5_H_4_), 4.11 (5H, s, C_5_H_5_), 4.13 (1H, m, C_5_H_4_), 4.15 (1H, m, C_5_H_4_), 4.24 (1H, m, C_5_H_4_), 4.27 (1H, m, C_5_H_4_), 4.80 (1H, m, C_5_H_4_), 4.85 (1H, m, C_5_H_4_), 4.41 (1H, m, CH), 7.07–7.19 (5H, m,C_6_H_5_), 7.59 (1H, s, CH=). ^13^C NMR [75 MHz, C_6_D_6_]: δ 42.38, 68.22 (2CH_2_), 68.10 (CH), 69.74, 70.06 (2C_5_H_5_), 68.76, 69.50 (2C), 70.72, 70.88, 71.06, 71.45, 71.59 (2C_5_H_4_), 81.45, 81.47 (2C_ipso_Fc), 126.68, 128.77, 129.63 (C_6_H_5_), 134.76 (CH=), 124.45, 138.87, 166.98 (3C). Anal. calcd. for C_32_H_29_Fe_2_NO: C, 69.22; H, 5.26; N, 2.52. Found: C, 69.43; H, 5.13; N, 2.65%. MS (El, 70 eV): *m*/*z* 555 [M]^+^. UV–vis (λ, nm): 328, 368, 462. E(I) = −65 mV, E(II) = 124 mV.

### 3.3. Computational Chemistry

Compound **3a** was optimized based on the molecular structure obtained by single crystal X-ray diffraction [36] using density functional theory with the M06-2x [57,58] level of theory and a 6-311G++(2d,2p) basis set. The functional has shown adequate results in evaluating diferrocenyl derivatives [36]. The optimized structure of **3a** was used to generate the rest of the molecules, performing a full optimization using the same method and basis set. Calculated frequencies showed no negative results, confirming the geometry optimization’s convergence. All the quantum mechanics calculations were performed using the D.01 version of the Gaussian program [59], and HOMO and LUMO orbitals were calculated and displayed using GaussView [60].

### 3.4. Amoebicidal Activity 

Trophozoites of the *E. histolytica* HM1:IMSS strain were axenically grown by placing 1 × 10^5^ live trophozoites in tubes with 3 mL of supplemented TYI-S33 medium. Amoebic trophozoite viability exposed to 1000, 100, 10, 1, 0.1, and 0.01 μM of each experimental compound was determined at 24, 48, and 72 h with trypan blue vital marker and confirmed with the fluorescent dyes carboxyfluorescein diacetate (CFDA) and propidium iodide. Either 100 μL of Trypan blue 0.4% or 1 μL of 5 μM CFDA (Invitrogen Molecular Probes, Inc., Eugene, OR, USA) and 1 μL of 1.5 μM propidium iodide were added to samples of 100 μL containing around 1 × 10^4^ treated trophozoites, mixed, and incubated at room temperature for 15 min. The final solutions were mixed and incubated at room temperature for 15 min to perform the counting with an Olympus BX51 fluorescence microscope. The IC_50_ values were calculated by a multivariable regression using the amoebic viability curves for the tree times evaluated on GraphPad Prism 10.0.

### 3.5. Determination of LogP by the TLC Method

LogP was determined using the reversed phase-thin layer chromatography (RP-TLC) described by Perjési and co-workers [42]. Aluminum plates (20 × 20) precoated with silica gel 60 TLC F_254_ were washed with methanol and dried. Diferrocenyl oxazole derivatives were dissolved in a 1:1 methanol/chloroform mixture with methanol/water 80:20 as the mobile phase. The chromatographic chamber was saturated with mobile phase for 30 min before use. After development, the plates were dried and revealed under UV light (λ = 254 nm) to determine the retention factor (RM). We used the equation logP = 4.7927R_M_ + 1.7897 previously reported by Perjési to describe ferrocenyl derivatives’ hydrophilic/lipophilic characteristics. Progesterone was used to validate the logP measurements. The logP value found for progesterone was 3.6. Three measurements were performed for each compound.

## 4. Conclusions

The results of this work and data compiled from the literature show a clear pipeline for designing more effective amoebicidal agents by fine-tuning the redox potential and hydrophilic properties of mono- and di-ferrocenyl products, tending to the redox potential of metronidazole but exploiting the same and new biological targets. The antiproliferative activity of 2-(*Z*-1,2-diferrocenylvinyl)-4,5-dihydrooxazole derivatives evaluated on HM1:IMSS trophozoites shows the need for a balance between redox potential and hydrophobic character to improve the antiproliferative activity of diferrocenyl derivatives studied herein. Compound **3d** presents the highest antiproliferative activity of the series with IC_50_ = 23.7 µM. This compound also has the lowest redox potential, the highest wavelength value for the LE electronic transition, and one of the highest logP values. The theoretical parameters obtained from the DFT study, such as the HOMO and Δ_HOMO–LUMO_, could be helpful in the design of highly effective amoebicidal agents in further studies.

## Figures and Tables

**Table 1:** ^1^H and ^13^C chemical shifts, redox potentials (E(I) and E(II)), comproportionation constant (Kcom), electronic transitions, logP, and frontier orbitals of compounds **3a**–**e**.

	H NMR	C NMR	E(I)	E(II)	Log K_com_	UV–vis	logP ^b^	HOMO	LUMO	Δ_HOMO–LUMO_
**3a**	7.15	136.94	0.116 ^a^	0.305 ^a^	3.19 ^a^ (1563)	458, 368, 330	4.42 (0.2)	−0.526	−0.357	−0.169
**3b**	7.14	134.20	−0.019 ^a^	0.182 ^a^	3.39 ^a^ (2495)	466, 372, 331	4.76 (0.1)	−0.515	−0.358	−0.156
**3c**	7.62	134.43	−0.022	0.167	1563	464, 368, 329	5.48 (0.1)	−0.511	−0.358	−0.152
**3d**	7.29	135.04	−0.045 ^a^	0.135 ^a^	1101 ^a^	468, 370, 334	5.62 (0.2)	−0.461	−0.354	−0.106
**3e**	7.59	134.76	−0.065	0.124	1557	462, 368, 328	6.1 (0.09)	−0.450	−0.358	−0.091

^a^ Redox potential values of compounds **1**, **2**, and **4** were taken from Reference [37]. ^b^ Values in parentheses are the standard deviations from three independent determinations.

**Table 2:** Amoebicidal activity of 2-(*Z*-1,2-diferrocenylvinyl)-4,5-dihydrooxazole derivatives **3a**–**e**. The half-inhibition concentration (IC_50_) is expressed in µM and logarithmic units (−log (1/IC_50_).

Compound	3a	3b	3c	3d	3e	Metronidazole
IC_50_ (µM)	1000 ± 46	100 ± 9	1891 ± 28	23 ± 3	385 ± 11	7 ± 0.9
−log (1/IC_50_)	3	2	3.27	1.36	2.58	−0.84

## Data Availability

The data derived from this work may be asked for by email from the corresponding authors.

## Sample Availability

Samples of the compounds can be requested from the authors.
